# Buffalo Medical Association

**Published:** 1845-08-01

**Authors:** 


*Buffalo Medical Association.*—An adjourned Meeting of the Physicians of this city was held on the 16th ultimo, and a Constitution and Bye-Laws for a City Association, reported by a committee appointed at a previous meeting. After some discussion, these were adopted, the Association regularly organized, and Officers for the year elected. The objects of the Association, as declared in the constitution, are, mutual improvement by the communication of facts, free discussion, and the interchange of views on subjects connected with medicine and the medical profession; the cultivation of social relations, and the regulation of professional intercourse by the enforcement of the practical application of the principles of medical ethics.

With the professed objects of the Association, none, surely, can find fault. They are not merely laudable, but of very great importance. It is, also, needless to say, that whether the present Association shall fulfil these objects or not, is for the medical fraternity here to determine. It *can* and *will* succeed, and produce good results, if we choose to unite in resolving that it *shall* be so.

The Officers chosen for the ensuing year are as follows: *President*—JOSIAH TROWBRIDGE M. D.; *Vice President*—ALDEN S. SPRAGUE, M. D.; *Recording Secretary*—AUSTIN FLINT, M. D. The Meetings are appointed for the *First Tuesday in each month*.

☞ The First Regular Meeting will be on Tuesday Evening, the 5th inst. at 8 P. M., at the office of F. H. HAMILTON, M. D.

